# The research progress and future directions in the pathophysiological mechanisms of type 2 diabetes mellitus from the perspective of precision medicine

**DOI:** 10.3389/fmed.2025.1555077

**Published:** 2025-03-05

**Authors:** Xinyi Tian, Liuqing Wang, Liuting Zhong, Kaiqi Zhang, Xiaolei Ge, Zhengrong Luo, Xu Zhai, Shaoyan Liu

**Affiliations:** ^1^School of Acupuncture-Moxibustion and Tuina, Shandong University of Traditional Chinese Medicine, Jinan, China; ^2^Institute of Chinese Medical History and Literatures, China Academy of Chinese Medical Sciences, Beijing, China; ^3^First School of Clinical Medicine, Anhui University of Traditional Chinese Medicine, Hefei, China; ^4^Wangjing Hospital, China Academy of Chinese Medical Sciences, Beijing, China; ^5^Graduate School, China Academy of Chinese Medical Sciences, Beijing, China

**Keywords:** type 2 diabetes mellitus, pathophysiology, metabolism, precision medicine, T2DM

## Abstract

Type 2 diabetes mellitus (T2DM) is a complex metabolic disorder characterized by pathophysiological mechanisms such as insulin resistance and *β*-cell dysfunction. Recent advancements in T2DM research have unveiled intricate multi-level regulatory networks and contributing factors underlying this disease. The emergence of precision medicine has introduced new perspectives and methodologies for understanding T2DM pathophysiology. A recent study found that personalized treatment based on genetic, metabolic, and microbiome data can improve the management of T2DM by more than 30%. This perspective aims to summarize the progress in T2DM pathophysiological research from the past 5 years and to outline potential directions for future studies within the framework of precision medicine. T2DM develops through the interplay of factors such as gut microbiota, genetic and epigenetic modifications, metabolic processes, mitophagy, NK cell activity, and environmental influences. Future research should focus on understanding insulin resistance, *β*-cell dysfunction, interactions between gut microbiota and their metabolites, and the regulatory roles of miRNA and genes. By leveraging artificial intelligence and integrating data from genomics, epigenomics, metabolomics, and microbiomics, researchers can gain deeper insights into the pathophysiological mechanisms and heterogeneity of T2DM. Additionally, exploring the combined effects and interactions of these factors may pave the way for more effective prevention strategies and personalized treatments for T2DM.

## 1 Introduction

T2DM, characterized by persistently elevated blood glucose levels, is increasingly recognized as a complex metabolic disease affecting the heart and kidneys (1, 2). It also represents a significant public health challenge, being the fastest-growing metabolic disease worldwide (3) and affecting approximately 10% of the global population (4). Over the past three decades, the number of diabetes cases has doubled globally. Currently, one in 11 adults is diagnosed with diabetes, and 90% of these cases are T2DM. This makes T2DM a rapidly growing global health concern (5). Asia, particularly China and India, is a key region for the rising prevalence of T2DM (6). While developed countries report higher prevalence rates (7), developing nations are experiencing faster growth in cases (8). The development of T2DM is closely associated with multiple risk factors. Obesity, often accompanied by hyperinsulinemia and insulin resistance, is one of the main contributors (9). While T2DM typically occurs in middle-aged and older adults, its incidence among adolescents and children has been increasing due to rising obesity rates in these populations (10, 11). Environmental factors, such as specific medications and chemicals, may also induce insulin resistance, thereby elevating the risk of T2DM (12). Genetic predisposition significantly impacts T2DM onset, as individuals with a family history of the disease have a higher risk (13). Additionally, other diseases, including thyroid disorders (14), Cushing’s syndrome (15), hypertension (16), and metabolic syndrome (17), are known to increase the risk of developing T2DM.The epidemiology of T2DM is therefore a complex and multifaceted issue. Addressing it requires comprehensive interventions, such as promoting healthy eating habits, increasing physical activity, improving healthcare access, and enhancing health education, which are crucial for reducing the prevalence and impact of T2DM.

Precision medicine, defined as “an emerging approach for disease treatment and prevention that considers individual variability in genes, environment, and lifestyle, “introduces a tailored approach to managing diseases (18). This perspective has shifted the understanding of T2DM from a single disease to a condition comprising multiple subtypes, each presenting distinct features in critical pathological processes such as insulin resistance and *β*-cell dysfunction (19). Identifying these subtypes at the diagnostic stage facilitates personalized treatment strategies, enabling clinical resources to focus on patients at the highest risk of complications. This approach not only enhances patient outcomes but also reduces healthcare costs (20). This emphasis on personalized care is supported by the treatment recommendations of the American Diabetes Association and the European Association for the Study of Diabetes, which highlight the need for patient-centered therapeutic approaches based on individual characteristics and comorbidities (21).

Advances in large-scale genomics and metabolomics analyses have identified key genes associated with insulin signaling pathways and *β*-cell health, as well as the significant role of epigenetic modifications in the development of T2DM (22). The gut microbiota and its metabolites have also been shown to influence glucose metabolism and insulin sensitivity, contributing to the pathogenesis of T2DM (23). In addition, mitophagy and NK cell activity are involved in maintaining systemic metabolic homeostasis and are linked to T2DM onset. Alongside these mechanisms, interactions among lipid metabolism, dietary patterns, and environmental factors further complicate the disease’s pathogenesis. Comprehensive studies integrating these aspects will enhance the understanding of T2DM’s pathophysiology and support the development of strategies for prevention and personalized treatment.

With the emergence of precision medicine in the field of T2DM, there has been a lack of prospective studies that comprehensively examine the advancements in T2DM pathophysiology over the past 5 years. Additionally, there has been limited exploration of the future research directions of T2DM pathophysiology through the lens of precision medicine, which would provide a foundation for targeted therapies and drug development. This perspective study focuses on the key recent findings in T2DM pathophysiology over the past 5 years and discusses the future directions of research on T2DM pathophysiological mechanisms within the precision medicine framework, incorporating multi-omics analysis and artificial intelligence technologies.

## 2 Recent advances in the pathophysiological mechanisms of T2DM over the past 5 years

### 2.1 Study design

The search of PubMed was conducted on 1 October 2024 since 1 January 2019, and included the broad terms ‘T2DM’[Title/Abstract],‘type 2 diabetes mellitus’[Title/Abstract]’,and‘pathophysiology’[Title/Abstract]’.Only peer reviewed English language publications were considered. The inclusion criteria included: a clinical randomized controlled trial (RCT) or animal experiment study; The exclusion criteria included: non-RCT, case analyses, literature reviews, retrospective analyses, meta-analyses and guidelines. The specific search strategy can be found in the Supplementary materials.

### 2.2 Gut microbiota and T2DM

A study published in May 2024 highlights the critical role of gut microbiota and its specificity in the pathogenesis of T2DM (24). Significant differences in the richness, composition, and function of microbial communities have been observed between individuals with and without T2DM (25). Research also suggests that gut bacteria associated with insulin resistance and sensitivity exhibit a unique carbohydrate metabolism pattern. This was demonstrated in mouse models, where the specific gut bacteria improved host insulin resistance phenotypes (26).

Moreover, several randomized controlled trials (RCTs) and animal studies have shown that the gut microbiota plays a pivotal role in T2DM progression. Metabolites derived from gut microbiota, such as bile acids, lipopolysaccharides, trimethylamine N-oxide (TMAO), tryptophan and indole derivatives, and short-chain fatty acids (SCFAs), have been implicated in the pathogenesis of T2DM and are key mediators in host-microbe crosstalk (27–30). Dysbiosis of gut microbiota in T2DM leads to abnormal gut metabolites and disruption of the intestinal barrier, promoting the translocation of bacteria and harmful metabolites into the circulatory system. These aberrant entries disrupt insulin sensitivity, glucose metabolism, and immune homeostasis, ultimately damaging multiple organs.

### 2.3 Genetics and epigenetic modifications in T2DM

Genome-wide association studies (GWAS) have identified over 240 loci associated with T2DM (31). For example, pathogenic heterozygous variants reducing the activity of the glucokinase (GCK) gene are linked to T2DM. Amino acid substitutions at active site residues directly influence protein activity, and critical residues involved in GCK’s metabolic stability and conformational dynamics regulate its activity, thereby affecting glucose homeostasis and contributing to T2DM development (32). These findings underscore the vital role of genetic variations in T2DM pathogenesis.

Epigenetic modifications, including DNA/RNA methylation, histone acetylation, and microRNA (miRNA) expression, are closely linked to T2DM onset (33). miRNAs, as “key regulators” in physiological and pathological processes, have become a prominent research topic. By regulating gene expression at transcriptional, post-transcriptional, and translational levels, miRNAs coordinate intercellular communication and regulate a broad range of cellular functions (34).

miRNAs are crucial regulators of insulin secretion (35), and their dysregulated expression is associated with the development and progression of T2DM (36). For instance, members of the miRNA-29 family, such as miR-29a-3p, miR-29b-3p, and miR-29c-3p, are widely expressed and upregulated under metabolic disease, obesity, and insulin resistance conditions. Maintaining a proper balance of miRNA-29 levels is critical for cellular and organ homeostasis in metabolism (37). Compared with non-diabetic individuals, T2DM patients show increased expression of miR-200c in pancreatic islets. Overexpression of miR-200c in EndoC-βH1 cells leads to reduced glucose-stimulated insulin secretion. The transcription factor ETV5 is a direct target of miR-200c in human islets. Knocking down miR-200c increases glucose-stimulated insulin secretion in islets from T2DM patients by approximately threefold, highlighting the significance of the miR-200c-ETV5 axis in *β*-cell dysfunction and T2DM pathophysiology (38). Compelling evidence suggests that miR-155 and miR-184-3p play pivotal roles in the pathogenesis of diabetes and its complications. Clinical studies report lower serum levels of miR-155 in T2DM patients. miR-155 is involved in phenotypic transitions of cells within the pancreatic islets under metabolic stress and modulates insulin sensitivity in the liver, adipose tissue, and skeletal muscle (39). miR-184-3p directly targets CREB and regulates the transcription coactivator CRTC1, protecting *β*-cells from lipotoxicity and inflammation-induced apoptosis. Its expression is reduced in the pancreatic islets of T2DM patients (40).

Additionally, the relationship between T2DM and DNA/RNA methylation has become a new research trend. T2DM and DNA/RNA methylation are highly dynamic and reversible processes that regulate gene expression. DNA methylation has been shown to maintain cellular metabolism and trigger *β*-cell dysfunction and insulin resistance, ultimately leading to T2DM. RNA methylation, in contrast, primarily regulates eukaryotic gene expression through post-transcriptional processes (41).

Compared to research on DNA/RNA methylation and miRNAs, studies on histone acetylation in T2DM are relatively limited. However, some evidence indicates that T2DM progression is associated with histone H3K9 and H3K23 acetylation, H3K4 monomethylation, and H3K9 dimethylation in the liver (42). Furthermore, histone H4 lysine 16 acetylation (H4K16ac) regulates central carbon metabolism in mice. Chronic imbalance in H4K16ac promotes metabolic instability, leading to the development of metabolic diseases (43).

### 2.4 Mitophagy and T2DM

Mitophagy, a selective autophagy process crucial for maintaining cellular homeostasis, plays a significant role in the pathogenesis of T2DM. It eliminates damaged and dysfunctional mitochondria, and substantial evidence indicates its importance in regulating T2DM mechanisms and maintaining metabolic stability. Defective mitophagy is closely associated with the development of insulin resistance (44).

A foundational experiment demonstrated that excessive mitophagy in adipose tissue disrupts systemic metabolic homeostasis by activating NF-κB signaling, linking it to hepatic insulin resistance and the onset of T2DM (45). Another study revealed that abnormal mitophagy mediated by the mitophagy receptor FUNDC1 in adipose tissue results in mitochondrial quality control dysregulation, increased oxidative stress, and overactivation of the MAPK pathway. These changes lead to white adipose tissue (WAT) remodeling characterized by enhanced infiltration of adipose tissue macrophages (ATMs), M1 macrophage polarization, and chronic inflammatory responses, all of which are closely linked to metabolic disorders (46). Among these, exosomes derived from M1-polarized macrophages (M1-Exos) mediate intercellular transfer of miR-212-5p, targeting the sirtuin2 gene and regulating the Akt/GSK-3*β*/β-catenin pathway in recipient β-cells, ultimately suppressing insulin secretion and contributing to T2DM pathogenesis (47).

### 2.5 Natural killer (NK) cells and T2DM

NK cells are integral to the inflammatory microenvironment, contributing to various obesity-related metabolic diseases and playing a role in T2DM pathogenesis through systemic inflammation regulation. In obesity, the proliferation and activation of NK cells in visceral adipose tissue (VAT) are critical in the mechanisms of insulin resistance and T2DM development (48). A foundational experiment revealed that the cytotoxic activity of NK cells correlates with T2DM progression. In T2DM mice, the cytotoxicity of NK cells gradually decreases as the disease advances. Notably, even during the obesity-induced prediabetic stage, NK cell cytotoxicity is impaired. High glucose conditions further suppress NK cell activity, with reduced cytotoxicity being a common feature of both T1DM and T2DM (49). Clinical studies also demonstrate a significant inverse relationship between NK cell activity and fasting blood glucose, glycated hemoglobin, and 2-h postprandial glucose levels (50).

### 2.6 Metabolism and T2DM

Metabolic factors also play a vital role in T2DM pathogenesis. A September 2024 study identified hypertriglyceridemia as a risk factor for T2DM, potentially promoting its mechanisms either directly or via elevated non-esterified fatty acids (NEFAs). Mild acute hypertriglyceridemia was shown to impair glucose tolerance, insulin sensitivity, and clearance directly, with selective and opposing effects on *β*-cell function depending on NEFA neutralization. These findings offer new biological insights into the mechanisms underlying insulin resistance and chronic hyperinsulinemia during T2DM progression (51).

Additionally, the twin-cycle hypothesis provides a novel perspective on T2DM causation, proposing that excessive fat accumulation in the liver and pancreas represents a reversible cause of T2DM (52). Excess liver fat contributes to an over-supply of fat to the pancreas, resulting in dysfunction in both organs (53). Liver fat content positively correlates with systemic metabolic disturbances and the severity of chronic complications in T2DM patients (54), underscoring the critical role of fat metabolism in T2DM development.

### 2.7 Diet, environmental factors, and T2DM

Environmental factors also significantly impact T2DM. Studies show that trace elements like zinc, selenium, and copper are associated with T2DM development. While moderate zinc levels regulate insulin receptors, extend insulin action, and promote a healthy lipid profile, excessive levels may induce oxidative stress and exert toxic effects, contributing to T2DM progression (55).

The relationship between metal exposure and T2DM risk is gaining attention. A clinical study identified SOD2 and ICAM1 as potential targets of lead-induced T2DM, providing novel insights into the biological effects and mechanisms of internal metal exposure on T2DM in the Chinese population (56).

In summary, T2DM development is a complex process involving multiple interconnected factors, including gut microbiota, genetics and epigenetic modifications, metabolism, mitophagy, immunity, diet, and environmental influences. The dynamic interactions among these factors shape the pathogenesis of T2DM. Comprehensive studies of these elements will enhance our understanding of T2DM mechanisms and pave the way for innovative strategies in prevention and personalized treatment.

## 3 Precision medicine and future research directions

In recent years, significant progress has been made in understanding the pathophysiological mechanisms of T2DM. However, its complexity and diversity continue to present challenges for precision medicine. The concept of precision medicine emphasizes the tailoring of treatment strategies based on an individual’s genes, environment, lifestyle, and the specific molecular mechanisms of the disease. From the perspective of precision medicine, future research on the pathophysiology of T2DM will increasingly focus on personalization, precision, and in-depth exploration at the molecular mechanism level (57). Based on current research advances, future studies on the pathophysiological mechanisms of T2DM from the precision medicine viewpoint can be directed toward the following five aspects.

### 3.1 Multi-omics analysis of T2DM pathophysiology

The pathophysiological mechanisms and clinical manifestations of T2DM patients are highly heterogeneous. Multi-omics analysis (including genomics, transcriptomics, proteomics, metabolomics, etc.) provides a more comprehensive perspective to explore the pathophysiological mechanisms of T2DM (58).

Genomic studies have identified several susceptibility genes associated with T2DM, such as TCF7L2 and PPARG, which are involved in insulin secretion and metabolic regulation. Transcriptomic analysis reveals gene expression abnormalities in the *β*-cells, adipose tissue, and liver of T2DM patients, particularly genes related to insulin secretion, inflammatory responses, and fat metabolism.

Moreover, proteomic analysis has shown significant changes in the expression of insulin signaling pathways, inflammatory factors, and metabolic enzymes, suggesting mechanisms of insulin resistance and *β*-cell dysfunction. Metabolomic studies, on the other hand, reveal abnormalities in metabolites such as glucose, fatty acids, and amino acids, reflecting disruptions in glucose and fat metabolism (59). Integrating multi-omics data allows for a more comprehensive understanding of the pathophysiology of T2DM, providing a new theoretical basis for personalized treatment and early intervention.

### 3.2 Applications of big data and artificial intelligence

From the perspective of precision medicine, the pathophysiology of T2DM can be deeply understood through the integration of multi-level information, including genes, metabolism, environment, and lifestyle. The goal of precision medicine is to develop personalized treatment plans based on individual characteristics, optimizing clinical outcomes and prevention strategies. Artificial intelligence (AI) plays a crucial role in this process. By integrating genetic, metabolic, environmental, and lifestyle data from patients, AI algorithms, with their powerful data-processing capabilities, can synthesize multi-dimensional patient information (60). These algorithms can also uncover potential pathophysiological mechanisms of T2DM from large datasets, assisting researchers in further exploring the complex mechanisms of disease development and providing new directions for future research (61). Additionally, AI can perform personalized risk assessments and predictions, aiding in the early identification of T2DM risks and supporting the development of individualized treatment plans. By enabling real-time monitoring of blood glucose levels, physical activity, and diet through smart devices, AI provides personalized health management recommendations, advancing T2DM treatment towards personalization and intelligence. Precision medicine combined with AI is expected to enhance early prevention, treatment efficacy, and patients’ quality of life (62).

### 3.3 In-depth analysis of insulin resistance and *β*-cell dysfunction

One of the core pathological mechanisms of T2DM is insulin resistance and β-cell dysfunction (63). Insulin resistance reduces the sensitivity of tissue cells to insulin, impairing glucose utilization and increasing the burden on β-cells. Future research will focus on further refining the interaction mechanisms between these two factors, including the key molecules and regulatory mechanisms in the insulin signaling pathway, particularly in different tissues such as the liver, adipose tissue, and skeletal muscle (64). On the other hand, the molecular mechanisms of *β*-cell dysfunction will also be a key research direction. The relationship between β-cell dysfunction and genetic and epigenetic modifications, immune and inflammatory responses, as well as autophagy and apoptosis, may become a major research focus in the future (65).

### 3.4 Gut microbiota and its metabolites

Studies have shown that gut microbiota plays a pivotal role in the complex metabolic network of T2DM. The diversity and composition of these microbes, along with their metabolites, such as short-chain fatty acids and bile acids, have profound effects on insulin sensitivity and *β*-cell function. These metabolites can directly influence host metabolic pathways and indirectly regulate related signaling pathways (30). Current cutting-edge research reveals that gut dysbiosis is not only a key factor in the development and progression of T2DM but also closely associated with the exacerbation of insulin resistance and increased systemic inflammation. Therefore, under the wave of precision medicine, future research should focus on how to utilize multi-omics data to explore the intrinsic link between individual gut microbiota characteristics and the pathogenesis of T2DM. This will provide evidence for uncovering new disease mechanisms, developing personalized treatment plans, and formulating prevention strategies, effectively reducing the incidence of T2DM (66).

### 3.5 Micro-ribonucleic acid and genes

In recent years, large-scale genome-wide association studies (GWAS) and high-precision whole-genome sequencing technologies have enabled scientists to identify multiple gene variants closely associated with T2DM, such as TCF7L2 (67), FTO (68), and PPARG (69). These gene variants not only directly regulate insulin secretion, sensitivity, and action but also drive the onset and progression of T2DM through complex interactions with environmental factors, lifestyle, and metabolic pathways (70). Notably, miRNAs, as key epigenetic regulators, finely modulate the expression of related genes, forming a complex feedback network with DNA methylation, histone modifications, and other epigenetic mechanisms, further stabilizing and specifying gene expression (71). Furthermore, specific miRNAs show significant differences in the plasma or tissue samples of T2DM patients, offering potential for early diagnosis and prognosis evaluation (72). By analyzing miRNA expression profiles, doctors can develop personalized treatment plans. Therefore, in-depth exploration of the interactions between genomics and epigenetics will undoubtedly provide new perspectives and strategies for revealing the full picture of T2DM pathophysiology and developing personalized therapeutic approaches (73).

## 4 Discussion

Looking ahead, under the influence of precision medicine, research on T2DM will place greater emphasis on individual differences in the disease and the integration of multi-dimensional data. Through integrated multi-omics analysis and the application of big data and AI, researchers will be able to more deeply uncover the pathophysiological mechanisms of T2DM (76). This will not only help develop personalized treatment plans but also fundamentally change the approach to T2DM treatment, making it more precise and effective.

However, precision medicine entails the interdisciplinary integration of fields such as genomics, data science, and clinical medicine. In resource-limited settings, challenges related to data collection, integration, sharing, and privacy protection may arise, both from technical and legal perspectives. The existing healthcare system may struggle to rapidly adapt to the demands of precision medicine, thereby hindering its widespread adoption and application. Furthermore, the imbalance between the supply and demand for advanced technologies and specialized professionals in relevant fields presents additional potential obstacles to the effective implementation of precision medicine.

## 5 Conclusion

In the future, precision medicine holds great promise for providing more effective treatment options to improve health. This advancement is largely driven by new technologies, particularly the expansion of knowledge regarding disease mechanisms through large-scale biomedical research. Diseases like T2DM, with their heterogeneous nature, have multifactorial etiologies. Therefore, implementing precision medicine in T2DM requires not only a better understanding of genomics but also the integration of other types of omics, including epigenomics, proteomics, metabolomics, and pharmacogenomics, into a comprehensive precision medicine model for T2DM (74).

Moreover, the combination of precision medicine and artificial intelligence (AI) has tremendous potential in elucidating the pathophysiological mechanisms of T2DM, personalizing treatments, enabling early prevention, and facilitating dynamic monitoring. Through big data analysis and deep learning, AI can significantly enhance the diagnosis, treatment, and management of T2DM, providing patients with more precise and personalized medical services. This novel healthcare model not only improves treatment outcomes but also reduces treatment costs, advancing the smart and personalized development of diabetes management (75).

## Data Availability

The original contributions presented in the study are included in the article/Supplementary material, further inquiries can be directed to the corresponding authors.

## References

[1] Ahmad E Lim S Lamptey R Webb DR Davies MJ. Type 2 diabetes. Lancet. (2022) 400:1803–20. doi: 10.1016/S0140-6736(22)01655-5, PMID: 36332637

[2] Gurung RL Zheng H Lee BTK Liu S Liu J-J Chan C. Proteomics profiling and association with cardiorenal complications in type 2 diabetes subtypes in Asian population. Diabetes Res Clin Pract. (2024) 214:111790. doi: 10.1016/j.diabres.2024.111790, PMID: 39059739

[3] Ling C Bacos K Rönn T. Epigenetics of type 2 diabetes mellitus and weight change - a tool for precision medicine? Nat Rev Endocrinol. (2022) 18:433–48. doi: 10.1038/s41574-022-00671-w, PMID: 35513492

[4] Taylor SI Yazdi ZS Beitelshees AL. Pharmacological treatment of hyperglycemia in type 2 diabetes. J Clin Invest. (2021) 131:142243. doi: 10.1172/JCI142243, PMID: 33463546 PMC7810496

[5] Pillon NJ Loos RJF Marshall SM Zierath JR. Metabolic consequences of obesity and type 2 diabetes: balancing genes and environment for personalized care. Cell. (2021) 184:1530–44. doi: 10.1016/j.cell.2021.02.012, PMID: 33675692 PMC9191863

[6] Zheng Y Ley SH Hu FB. Global aetiology and epidemiology of type 2 diabetes mellitus and its complications. Nat Rev Endocrinol. (2018) 14:88–98. doi: 10.1038/nrendo.2017.151, PMID: 29219149

[7] Goodall R Alazawi A Hughes W Bravis V Salciccioli JD Marshall DC. Trends in type 2 diabetes mellitus disease burden in European Union countries between 1990 and 2019. Sci Rep. (2021) 11:15356. doi: 10.1038/s41598-021-94807-z, PMID: 34321515 PMC8319179

[8] Yoon K-H Lee J-H Kim J-W Cho JH Choi Y-H Ko S-H. Epidemic obesity and type 2 diabetes in Asia. Lancet. (2006) 368:1681–8. doi: 10.1016/S0140-6736(06)69703-1, PMID: 17098087

[9] Kahn SE Hull RL Utzschneider KM. Mechanisms linking obesity to insulin resistance and type 2 diabetes. Nature. (2006) 444:840–6. doi: 10.1038/nature05482, PMID: 17167471

[10] Magliano DJ Sacre JW Harding JL Gregg EW Zimmet PZ Shaw JE. Young-onset type 2 diabetes mellitus - implications for morbidity and mortality. Nat Rev Endocrinol. (2020) 16:321–31. doi: 10.1038/s41574-020-0334-z, PMID: 32203408

[11] Chandrasekaran P Weiskirchen R. The role of obesity in type 2 diabetes mellitus-An overview. Int J Mol Sci. (2024) 25:1882. doi: 10.3390/ijms25031882, PMID: 38339160 PMC10855901

[12] Li P Xu Y Li Z Cheng X Jia C Zhang S. Association between polychlorinated biphenyls exposure and incident type 2 diabetes mellitus: a nested case-control study. Environ Res. (2023) 228:115743. doi: 10.1016/j.envres.2023.115743, PMID: 37001846

[13] Ling C Rönn T. Epigenetics in human obesity and type 2 diabetes. Cell Metab. (2019) 29:1028–44. doi: 10.1016/j.cmet.2019.03.009, PMID: 30982733 PMC6509280

[14] Laclaustra M Moreno-Franco B Lou-Bonafonte JM Mateo-Gallego R Casasnovas JA Guallar-Castillon P. Impaired sensitivity to thyroid hormones is associated with diabetes and metabolic syndrome. Diabetes Care. (2019) 42:303–10. doi: 10.2337/dc18-1410, PMID: 30552134

[15] Krarup T Krarup T Hagen C. Do patients with type 2 diabetes mellitus have an increased prevalence of Cushing’s syndrome? Diabetes Metab Res Rev. (2012) 28:219–27. doi: 10.1002/dmrr.2262, PMID: 22162117

[16] Tanaka A Node K. Pathogenic connection between hypertension and type 2 diabetes: how do they mutually affect each other? Hypertens Res. (2022) 45:1840–2. doi: 10.1038/s41440-022-01014-y, PMID: 36100673

[17] Hudish LI Reusch JE Sussel L. β cell dysfunction during progression of metabolic syndrome to type 2 diabetes. J Clin Invest. (2019) 129:4001–8. doi: 10.1172/JCI129188, PMID: 31424428 PMC6763241

[18] Chung WK Erion K Florez JC Hattersley AT Hivert M-F Lee CG. Precision medicine in diabetes: a consensus report from the American Diabetes Association (ADA) and the European Association for the Study of diabetes (EASD). Diabetes Care. (2020) 43:1617–35. doi: 10.2337/dci20-0022, PMID: 32561617 PMC7305007

[19] Mizani MA Dashtban A Pasea L Zeng Q Khunti K Valabhji J. Identifying subtypes of type 2 diabetes mellitus with machine learning: development, internal validation, prognostic validation and medication burden in linked electronic health records in 420 448 individuals. BMJ Open Diabetes Res Care. (2024) 12:e004191. doi: 10.1136/bmjdrc-2024-004191, PMID: 38834334 PMC11163636

[20] Li L Cheng W-Y Glicksberg BS Gottesman O Tamler R Chen R. Identification of type 2 diabetes subgroups through topological analysis of patient similarity. Sci Transl Med. (2015) 7:311ra174. doi: 10.1126/scitranslmed.aaa9364, PMID: 26511511 PMC4780757

[21] Buse JB Wexler DJ Tsapas A Rossing P Mingrone G Mathieu C. A consensus report by the American Diabetes Association (ADA) and the European Association for the Study of diabetes (EASD). Diabetes Care. (2018) 43:487–93. doi: 10.2337/dci19-0066, PMID: 31857443 PMC6971782

[22] Lawlor N Stitzel ML. (epi)genomic heterogeneity of pancreatic islet function and failure in type 2 diabetes. Mol Metab. (2019) 27:S15–24. doi: 10.1016/j.molmet.2019.06.002, PMID: 31500827 PMC6768501

[23] García-Díez E López-Oliva ME Perez-Vizcaino F Pérez-Jiménez J Ramos S Martín MÁ. Dietary supplementation with a cocoa-carob blend modulates gut microbiota and prevents intestinal oxidative stress and barrier dysfunction in Zucker diabetic rats. Antioxidants (Basel). (2023) 12:1519. doi: 10.3390/antiox12081519, PMID: 37627514 PMC10452029

[24] Mei Z Wang F Bhosle A Dong D Mehta R Ghazi A. Strain-specific gut microbial signatures in type 2 diabetes identified in a cross-cohort analysis of 8,117 metagenomes. Nat Med. (2024) 30:2265–76. doi: 10.1038/s41591-024-03067-7, PMID: 38918632 PMC11620793

[25] Ding H Xu Y Cheng Y Zhou H Dong S Wu J. Gut microbiome profile of Chinese hypertension patients with and without type 2 diabetes mellitus. BMC Microbiol. (2023) 23:254. doi: 10.1186/s12866-023-02967-x, PMID: 37689641 PMC10492291

[26] Takeuchi T Kubota T Nakanishi Y Tsugawa H Suda W Kwon AT-J. Gut microbial carbohydrate metabolism contributes to insulin resistance. Nature. (2023) 621:389–95. doi: 10.1038/s41586-023-06466-x, PMID: 37648852 PMC10499599

[27] Zhou Z Sun B Yu D Zhu C. Gut microbiota: An important player in type 2 diabetes mellitus. Front Cell Infect Microbiol. (2022) 12:834485. doi: 10.3389/fcimb.2022.834485, PMID: 35242721 PMC8886906

[28] Ma Q Li Y Li P Wang M Wang J Tang Z. Research progress in the relationship between type 2 diabetes mellitus and intestinal flora. Biomed Pharmacother. (2019) 117:109138. doi: 10.1016/j.biopha.2019.109138, PMID: 31247468

[29] Wu J Yang K Fan H Wei M Xiong Q. Targeting the gut microbiota and its metabolites for type 2 diabetes mellitus. Front Endocrinol (Lausanne). (2023) 14:1114424. doi: 10.3389/fendo.2023.1114424, PMID: 37229456 PMC10204722

[30] Du L Li Q Yi H Kuang T Tang Y Fan G. Gut microbiota-derived metabolites as key actors in type 2 diabetes mellitus. Biomed Pharmacother. (2022) 149:112839. doi: 10.1016/j.biopha.2022.112839, PMID: 35325852

[31] Spracklen CN Horikoshi M Kim YJ Lin K Bragg F Moon S. Identification of type 2 diabetes loci in 433,540 east Asian individuals. Nature. (2020) 582:240–5. doi: 10.1038/s41586-020-2263-3, PMID: 32499647 PMC7292783

[32] Gersing S Schulze TK Cagiada M Stein A Roth FP Lindorff-Larsen K. Characterizing glucokinase variant mechanisms using a multiplexed abundance assay. Genome Biol. (2024) 25:98. doi: 10.1186/s13059-024-03238-2, PMID: 38627865 PMC11021015

[33] Suárez R Chapela SP Álvarez-Córdova L Bautista-Valarezo E Sarmiento-Andrade Y Verde L. Epigenetics in obesity and diabetes mellitus: new insights. Nutrients. (2023) 15:811. doi: 10.3390/nu15040811, PMID: 36839169 PMC9963127

[34] Lee W. MicroRNA, insulin resistance, and metabolic disorders. Int J Mol Sci. (2022) 23:16215. doi: 10.3390/ijms232416215, PMID: 36555853 PMC9785015

[35] Wendt A Esguerra JL Eliasson L. Islet microRNAs in health and type-2 diabetes. Curr Opin Pharmacol. (2018) 43:46–52. doi: 10.1016/j.coph.2018.08.003, PMID: 30144686

[36] Yu J Su W Zhang X Zheng F Guan Y. MicroRNAs in type 2 diabetes mellitus: important for the pathogenesis but uncertain as biomarkers. J Diabetes. (2020) 12:697–700. doi: 10.1111/1753-0407.12772, PMID: 29845732

[37] Dalgaard LT Sørensen AE Hardikar AA Joglekar MV. The microRNA-29 family: role in metabolism and metabolic disease. Am J Physiol Cell Physiol. (2022) 323:C367–77. doi: 10.1152/ajpcell.00051.2022, PMID: 35704699

[38] Ofori JK Karagiannopoulos A Nagao M Westholm E Ramadan S Wendt A. Human islet MicroRNA-200c is elevated in type 2 diabetes and targets the transcription factor ETV5 to reduce insulin secretion. Diabetes. (2022) 71:275–84. doi: 10.2337/db21-0077, PMID: 34753799 PMC8914283

[39] Jankauskas SS Gambardella J Sardu C Lombardi A Santulli G. Functional role of miR-155 in the pathogenesis of diabetes mellitus and its complications. Noncoding RNA. (2021) 7:39. doi: 10.3390/ncrna7030039, PMID: 34287359 PMC8293470

[40] Grieco GE Brusco N Fignani D Nigi L Formichi C Licata G. Reduced miR-184-3p expression protects pancreatic β-cells from lipotoxic and proinflammatory apoptosis in type 2 diabetes via CRTC1 upregulation. Cell Death Discov. (2022) 8:340. doi: 10.1038/s41420-022-01142-x, PMID: 35906204 PMC9338237

[41] Jiang C Hu Y Wang S Chen C. Emerging trends in DNA and RNA methylation modifications in type 2 diabetes mellitus: a bibliometric and visual analysis from 1992 to 2022. Front Endocrinol (Lausanne). (2023) 14:1145067. doi: 10.3389/fendo.2023.1145067, PMID: 37201099 PMC10187586

[42] Tu P Huang B Li M Zhang Y Bao S Tu N. Exendin-4 may improve type 2 diabetes by modulating the epigenetic modifications of pancreatic histone H3 in STZ-induced diabetic C57BL/6 J mice. J Physiol Biochem. (2022) 78:51–9. doi: 10.1007/s13105-021-00835-8, PMID: 34410626

[43] Pessoa Rodrigues C Chatterjee A Wiese M Stehle T Szymanski W Shvedunova M. Histone H4 lysine 16 acetylation controls central carbon metabolism and diet-induced obesity in mice. Nat Commun. (2021) 12:6212. doi: 10.1038/s41467-021-26277-w, PMID: 34707105 PMC8551339

[44] Shan Z Fa WH Tian CR Yuan CS Jie N. Mitophagy and mitochondrial dynamics in type 2 diabetes mellitus treatment. Aging (Albany NY). (2022) 14:2902–19. doi: 10.18632/aging.203969, PMID: 35332108 PMC9004550

[45] He F Huang Y Song Z Zhou HJ Zhang H Perry RJ. Mitophagy-mediated adipose inflammation contributes to type 2 diabetes with hepatic insulin resistance. J Exp Med. (2021) 218:e20201416. doi: 10.1084/jem.20201416, PMID: 33315085 PMC7927432

[46] Wu H Wang Y Li W Chen H Du L Liu D. Deficiency of mitophagy receptor FUNDC1 impairs mitochondrial quality and aggravates dietary-induced obesity and metabolic syndrome. Autophagy. (2019) 15:1882–98. doi: 10.1080/15548627.2019.1596482, PMID: 30898010 PMC6844496

[47] Qian B Yang Y Tang N Wang J Sun P Yang N. M1 macrophage-derived exosomes impair beta cell insulin secretion via miR-212-5p by targeting SIRT2 and inhibiting Akt/GSK-3β/β-catenin pathway in mice. Diabetologia. (2021) 64:2037–51. doi: 10.1007/s00125-021-05489-1, PMID: 34117507

[48] Li Y Wang F Imani S Tao L Deng Y Cai Y. Natural killer cells: friend or foe in metabolic diseases? Front Immunol. (2021) 12:614429. doi: 10.3389/fimmu.2021.614429, PMID: 33717101 PMC7943437

[49] Yoon Kim D Kwon LJ. Type 1 and 2 diabetes are associated with reduced natural killer cell cytotoxicity. Cell Immunol. (2022) 379:104578. doi: 10.1016/j.cellimm.2022.104578, PMID: 35908302

[50] Kim JH Park K Lee SB Kang S Park JS Ahn CW. Relationship between natural killer cell activity and glucose control in patients with type 2 diabetes and prediabetes. J Diabetes Investig. (2019) 10:1223–8. doi: 10.1111/jdi.13002, PMID: 30618112 PMC6717814

[51] Tricò D Rebelos E Astiarraga B Baldi S Scozzaro T Sacchetta L. Effects of hypertriglyceridemia with or without NEFA elevation on β-cell function and insulin clearance and sensitivity. J Clin Endocrinol Metab. (2024) 110:e667–74. doi: 10.1210/clinem/dgae276, PMID: 38635405 PMC11918624

[52] Taylor R Al-Mrabeh A Sattar N. Understanding the mechanisms of reversal of type 2 diabetes. Lancet Diabetes Endocrinol. (2019) 7:726–36. doi: 10.1016/S2213-8587(19)30076-2, PMID: 31097391

[53] Taylor R. Type 2 diabetes and remission: practical management guided by pathophysiology. J Intern Med. (2021) 289:754–70. doi: 10.1111/joim.13214, PMID: 33289165 PMC8247294

[54] Ren W Feng Y Feng Y Li J Zhang C Feng L. Relationship of liver fat content with systemic metabolism and chronic complications in patients with type 2 diabetes mellitus. Lipids Health Dis. (2023) 22:11. doi: 10.1186/s12944-023-01775-6, PMID: 36694216 PMC9872378

[55] Bjørklund G Dadar M Pivina L Doşa MD Semenova Y Aaseth J. The role of zinc and copper in insulin resistance and diabetes mellitus. Curr Med Chem. (2020) 27:6643–57. doi: 10.2174/0929867326666190902122155, PMID: 31475889

[56] Wang Y Shi P Zhao C Shi J Qi Z Xu S. Identification of the regulatory network and potential markers for type 2 diabetes mellitus related to internal exposure to metals in Chinese adults. Environ Geochem Health. (2023) 45:6889–902. doi: 10.1007/s10653-023-01504-z, PMID: 36811699

[57] Gloyn AL Drucker DJ. Precision medicine in the management of type 2 diabetes. Lancet Diabetes Endocrinol. (2018) 6:891–900. doi: 10.1016/S2213-8587(18)30052-4, PMID: 29699867

[58] Hwang Y-C Ahn H-Y Jun JE Jeong I-K Ahn KJ Chung HY. Subtypes of type 2 diabetes and their association with outcomes in Korean adults - a cluster analysis of community-based prospective cohort. Metabolism. (2023) 141:155514. doi: 10.1016/j.metabol.2023.155514, PMID: 36746321

[59] Ahlqvist E Prasad RB Groop L. 100 YEARS OF INSULIN: towards improved precision and a new classification of diabetes mellitus. J Endocrinol. (2021) 252:R59–70. doi: 10.1530/JOE-20-0596, PMID: 34783681

[60] Tanabe H Sato M Miyake A Shimajiri Y Ojima T Narita A. Machine learning-based reproducible prediction of type 2 diabetes subtypes. Diabetologia. (2024) 67:2446–58. doi: 10.1007/s00125-024-06248-8, PMID: 39168869 PMC11519166

[61] Mosquera-Lopez C Jacobs PG. Digital twins and artificial intelligence in metabolic disease research. Trends Endocrinol Metab. (2024) 35:549–57. doi: 10.1016/j.tem.2024.04.019, PMID: 38744606

[62] Ding J-E Thao PNM Peng W-C Wang J-Z Chug C-C Hsieh M-C. Large language multimodal models for new-onset type 2 diabetes prediction using five-year cohort electronic health records. Sci Rep. (2024) 14:20774. doi: 10.1038/s41598-024-71020-2, PMID: 39237580 PMC11377777

[63] Stumvoll M Goldstein BJ van Haeften TW. Type 2 diabetes: principles of pathogenesis and therapy. Lancet. (2005) 365:1333–46. doi: 10.1016/S0140-6736(05)61032-X, PMID: 15823385

[64] Christensen AA Gannon M. The Beta cell in type 2 diabetes. Curr Diab Rep. (2019) 19:81. doi: 10.1007/s11892-019-1196-4, PMID: 31399863

[65] Berbudi A Rahmadika N Tjahjadi AI Ruslami R. Type 2 diabetes and its impact on the immune system. Curr Diabetes Rev. (2020) 16:442–9. doi: 10.2174/1573399815666191024085838, PMID: 31657690 PMC7475801

[66] Wu H Tremaroli V Schmidt C Lundqvist A Olsson LM Krämer M. The gut microbiota in prediabetes and diabetes: a population-based cross-sectional study. Cell Metab. (2020) 32:379–390.e3. doi: 10.1016/j.cmet.2020.06.011, PMID: 32652044

[67] Zeini M Laurenti MC Egan AM Muthusamy K Ramar A Vella E. The longitudinal effect of diabetes-associated variation in TCF7L2 on islet function in humans. Diabetes. (2024) 73:1440–6. doi: 10.2337/db24-0192, PMID: 38869455 PMC11333375

[68] Hussain M Waheed A Elahi A Mustafa G. Fat mass and obesity-related (FTO) gene variant is a predictor of CVD in T2DM patients. J Diabetes Res. (2024) 2024:5914316. doi: 10.1155/2024/5914316, PMID: 39257882 PMC11383650

[69] da Silva MA Soares RMV de Oliveira Filho AF Campos LRS de Lima JG de Melo Campos JTA. Case report: two novel PPARG pathogenic variants associated with type 3 familial partial lipodystrophy in Brazil. Diabetol Metab Syndr. (2024) 16:145. doi: 10.1186/s13098-024-01387-9, PMID: 38951919 PMC11218129

[70] Laakso M Fernandes SL. Genetics of type 2 diabetes: past, present, and future. Nutrients. (2022) 14:3201. doi: 10.3390/nu14153201, PMID: 35956377 PMC9370092

[71] Emantoko Dwi Putra S Martriano Humardani F Antonius Y Jonathan J Thalia ML. Epigenetics of diabetes: a bioinformatic approach. Clin Chim Acta. (2024) 557:117856. doi: 10.1016/j.cca.2024.117856, PMID: 38490340

[72] Grieco GE Besharat ZM Licata G Fignani D Brusco N Nigi L. Circulating microRNAs as clinically useful biomarkers for type 2 diabetes mellitus: miRNomics from bench to bedside. Transl Res. (2022) 247:137–57. doi: 10.1016/j.trsl.2022.03.008, PMID: 35351622

[73] Udler MS Kim J von Grotthuss M Bonàs-Guarch S Cole JB Chiou J. Type 2 diabetes genetic loci informed by multi-trait associations point to disease mechanisms and subtypes: a soft clustering analysis. PLoS Med. (2018) 15:e1002654. doi: 10.1371/journal.pmed.1002654, PMID: 30240442 PMC6150463

[74] Krook A Mulder H. Pinpointing precision medicine for diabetes mellitus. Diabetologia. (2022) 65:1755–7. doi: 10.1007/s00125-022-05777-4, PMID: 35997779

[75] Lin Y Wessel J. The continuing evolution of precision health in type 2 diabetes: achievements and challenges. Curr Diab Rep. (2019) 19:16. doi: 10.1007/s11892-019-1137-2, PMID: 30806820

[76] Wang G Chiou J Zeng C Miller M Matta I Han JY. Integrating genetics with single-cell multiomic measurements across disease states identifies mechanisms of beta cell dysfunction in type 2 diabetes. Nat Genet. (2023) 55:984–94. doi: 10.1038/s41588-023-01397-9, PMID: 37231096 PMC10550816

