# Supplementary material for: The research progress and future directions in the pathophysiological mechanisms of type 2 diabetes mellitus from the perspective of precision medicine
Source: Front Med (Lausanne). 2025 Mar 5;12:1555077. doi: 10.3389/fmed.2025.1555077 (PMC11919862; doi:10.3389/fmed.2025.1555077)
Supplement: Supplementary file 1 [file Table_1.docx]

# Supplementary Material

| Items | Specification |
| --- | --- |
| Date of search | October 1,2024 |
| Databases and other sources searched | PubMed |
| Search terms used | “type 2 diabetes mellitus”[Title/Abstract] “T2DM”[Title/Abstract] “T2D” [Title/Abstract] “type 2 diabetes” [Title/Abstract] AND “pathophysiology” [Title/Abstract] “pathology” [Title/Abstract] “physiology” [Title/Abstract] “physiopathology” [Title/Abstract] |
| Search term example | ((T2DM) OR (T2D) OR (type 2 diabetes mellitus) OR (type 2 diabetes)) AND ((pathophysiology) OR (pathology) OR (physiology) OR (physiopathology)) |
| Timeframe | 1 October 2024 - 1 January 2019 |
| Selection process | Inclusion criteria included:   1. peer eviewed english language publications; 2. clinical randomized controlled trial (RCT) ; 3. animal experiment study. Exclusion criteria included:    1. non-RCT;    2. case analyses;    3. literature reviews,retrospective analyses, meta-analyses and guidelines. |
