# Supplementary material for: The research progress and future directions in the pathophysiological mechanisms of type 2 diabetes mellitus from the perspective of precision medicine
Source: Front Med (Lausanne). 2025 Mar 5;12:1555077. doi: 10.3389/fmed.2025.1555077 (PMC11919862; doi:10.3389/fmed.2025.1555077)
Supplement: Supplementary file 2 [file Table_2.docx]

| **pathophysiology** | | **Study** | **Category** | | **Outcomes** |
| --- | --- | --- | --- | --- | --- |
| **Gut Microbiota** | Mei, Z., et al., | Adults | Strain-specific gut microbial signatures in T2DM identified in a cross-cohort analysis of 8,117 metagenomes. | |  |
|  | Ding, H., et al., | Adults | There were significant differences in microbial community richness, composition and function between healthy controls, hypertensive patients and those without T2DM. | |  |
|  | Takeuchi, T., et al., | Adults | Fecal carbohydrates are increased in insulin-resistant individuals and are associated with microbial carbohydrate metabolism and host inflammatory cytokines. | |  |
|  | Zhou Z., et al., | Rats | Molecular mechanisms linking host and gut microbiota in T2DM include host molecules that induce gut microbiota dysregulation, immune and inflammatory responses, and gut microbial metabolites involved in pathogenesis. | |  |
|  | Du L., et al., | Rats and Adults | Gut microbiome derived metabolites are key factors in host-microbial crosstalk and play a central role in the physiology and physiological pathology of T2DM. | |  |
| **Genetics and Epigenetic Modification** | Gersing, S., et al., | Adults | The mechanism basis of low-activity variants was identified by identifying variants that affect the stability and conformational dynamics of GCK proteins and the residues that regulate the stability and dynamics were identified. | |  |
|  | Dalgaard, L.T., et al., | Rats | miR-29 family mirnas play an important role in various organs related to intermediate metabolism, and their upregulation leads to impaired glucose metabolism and inhibits fibrosis development. | |  |
|  | Ofori, J.K., et al., | Adults | The miR-200c-ETV5 axis plays an important role in T2D beta cell dysfunction and pathophysiology. | |  |
|  | Grieco, G.E., et al., | Adults | Decreased expression of NKX6.1 was accompanied by a significant decrease in expression of miR-184-3p, and the reduction of miR-184-3p protected beta cells from apoptosis through a CRTC1-dependent mechanism. | |  |
|  | Tu, P., et al., | Rats | Exendin-4 may improve T2DM by modulating the epigenetic modifications of pancreatic histone H3 in STZ-induced diabetic C57BL/6 J mice. | |  |
|  | Pessoa Rodrigue., et al., | Rats | Histone H4 lysine 16 acetylation controls central carbon metabolism and diet-induced obesity in mice. | |  |
| **Mitophagy** | He, F., et al., | Rats | Rats with elevated mitochondrial reactive oxygen species (ROS) pathways and NF-κB signaling and altered fatty acid metabolism in T2DM adipocytes with lipospecific loss of REDOX Trx2 developed hyperglycemia, hepatic insulin resistance and hepatic steatosis. | |  |
|  | Wu, H., et al., | Rats | Deficiency of mitophagy receptor FUNDC1 impairs mitochondrial quality and aggravates dietary-induced obesity and metabolic syndrome. | |  |
|  | Qian, B., et al., | Rats | M1 macrophage-derived exosomes impair beta cell insulin secretion via miR-212-5p by targeting SIRT2 and inhibiting Akt/GSK-3β/β-catenin pathway in mice. | |  |
| **Natural Killer Cells** | Yoon D., et al., | Rats | High glucose condition can affect NK cell activity, Type 1 and 2 diabetes are associated with reduced natural killer cell cytotoxicity. | |  |
|  | Kim, J.H., et al., | Adults | Compared with individuals with normal glucose tolerance or prediabetes, NK cell activity was reduced in T2DM, which was significantly associated with glycemic control. | |  |
|  | Wang H., et al., | Adults | Tim-3 expression on NK cells is inversely correlated with TNF-α production, and NK cell dysfunction with enhanced Tim-3 expression occurs in T2DM patients and is associated with their increased susceptibility to cancer and infectious diseases | |  |
|  | Dai C., et al., | Adults | ABHD17A and NPEPPS are key genes in the coexistence of CAD and T2DM, CD8(+) T and NK cells characterized by upregulation of NPEPPS and ABHD17A are associated with the co-occurrence of T2DM and coronary artery disease. | |  |
| **Metabolism** | Tricò D., et al., | Adults | Mild acute hypertriglyceridemia directly reduces glucose tolerance, insulin sensitivity, and clearance, and has selective and opposite effects on beta cell function neutralized by NEFAs. | |  |
|  | Ren W., et al., | Adults | Diabetes course, TC, HOMA-IR and LFC are risk factors for ASCVD complications, while diabetes course, TG and LDL-C are risk factors for DN complications. In addition, duration of diabetes and SBP are risk factors for complications of DR And DPN in T2DM patients. | |  |
|  | Al-Mrabeh A., et al., | Adults | Weight-related fat metabolism disorders such as VLDL1-triglyceride production, hepatic palmitic acid flux, and pancreatic fat appear to drive the development and reversal of T2DM. | |  |
| **Diet and Environmental Factors** | Bjørklund G., et al., | Adults | Zinc appears to activate key molecules involved in cell signaling, thereby maintaining glucose homeostasis. Zinc also regulates insulin receptors, prolongs the action of insulin, and promotes a healthy lipid profile. Excess copper produces oxidative stress. | |  |
|  | Wang Y., et al., | Adults | SOD2 and ICAM1 showed strong similarity in expression, indicating a functional correlation between these key genes. SOD2 and ICAM1 may be potential targets for Pb expose-induced T2DM. | |  |
|  | Liu J ., et al., | Adults | Serum Mn, Zn, Mo, Ba, Lu, Hg, Tl, and Pb were associated with T2D risk. Two metabolites mediate the association between serum Pb concentration and T2D risk. | |  |
